# Supplementary material for: Tracking receptor motions at the plasma membrane reveals distinct effects of ligands on CCR5 dynamics depending on its dimerization status
Source: eLife. 2022 Jul 22;11:e76281. doi: 10.7554/eLife.76281 (PMC9307273; doi:10.7554/eLife.76281)
Supplement: Figure 1—source code 1. [file elife-76281-fig1-code1.zip › Matlab code/Description.docx]

The 2 main Matlab function are “MAIN_cellbycell.m” for classifying experimental tracks and “MAIN_MOTIONsimu” that simulates Brownian/Confined & Directed trajectories and classifies simulated trajectories with our statistical framework or MSD analysis.

*Main output of “MAIN_cellbycell.m” are .eps figures*

- ***filename-cell-i-(withallimmo)-classification.eps*** contains the color plot of experimental trajectories & the overall result of classification (% of Brownian (red), Confined (blue), Directed (green) & Immobile (black) tracks) for cell number *i* (corresponding to the *i^th^* sheet of the data excel file (DATA/filename.xls), with or without Immobile tracks (see Material & Methods for the definition of immobile tracks)

***- filename -cell-i-NbChanges-mob.eps*** is the bar graph of the % of entiley Brownian/Confined/Restricted/Immobile tracks

*Main output of “MAIN_MOTIONsimu.m” are .eps figures of*

***- (brownian/confined/directed)_tracks.eps:*** Simulated Brownian/Confined & Directed tracks

***-(brownian/confined/directed)Stat_tracks.eps:*** color plot of simulated Brownian/Confined & Directed trajectories & the overall result of classification (% of Brownian (red), Confined (blue), Directed (green) & Immobile (black) tracks) using our statistical framework

***-(brownian/confined/directed)MSD_tracks.eps:*** color plot of simulated Brownian/Confined & Directed trajectories & the overall result of classification (% of Brownian (red), Confined (blue), Directed (green) & Immobile (black) tracks) using our MSD classification.

**DATA**

To test the code, we provided an excel file (Classeur_track S1_example) in the DATA folder. In this file, each sheet contains the tracks (generated with ICY spot tracking plugin) corresponding to different cells of the same sample. Sheets 1 to 5: untreated sample, sheets 6 to 9: sample treated with PSC-RANTES (20nM) at different times from 1 to 4 min.
